# Perfect match: mTOR inhibitors and tuberous sclerosis complex

**DOI:** 10.1186/s13023-022-02266-0

**Published:** 2022-03-04

**Authors:** Cong Luo, Wen-Rui Ye, Wei Shi, Ping Yin, Chen Chen, Yun-Bo He, Min-Feng Chen, Xiong-Bin Zu, Yi Cai

**Affiliations:** 1 Department of Urology, Disorders of Tuberous Sclerosis Complex (TSC) Multidisciplinary Team, National Clinical Research Center for Geriatric Disorders, Xiangya Hospital, Central South University, Changsha City, 410008 Hunan Province People’s Republic of China; 2 Department of Neurosurgery, Xiangya Hospital, Central South University, No. 87 Xiangya Road, Changsha City, 410008 Hunan Province People’s Republic of China; 3 Department of Dermatology, Disorders of Tuberous Sclerosis Complex (TSC) Multidisciplinary Team, Xiangya Hospital, Central South University, No. 87 Xiangya Road, Changsha City, 410008 Hunan Province People’s Republic of China; 4 Department of Oral and Maxillofacial Surgery, Center of Stomatology, Disorders of Tuberous Sclerosis Complex (TSC) Multidisciplinary Team, Xiangya Hospital, Central South University, No. 87 Xiangya Road, Changsha City, 410008 Hunan Province People’s Republic of China; 5 Department of Pediatrics, Disorders of Tuberous Sclerosis Complex (TSC) Multidisciplinary Team, National Clinical Research Center for Geriatric Disorders, Xiangya Hospital, Central South University, No. 87 Xiangya Road, Changsha City, 410008 Hunan Province People’s Republic of China

**Keywords:** Tuberous sclerosis complex (TSC), mTOR inhibitors, Efficacy, Adverse events, Precision medicine

## Abstract

Tuberous sclerosis complex (TSC) is an autosomal dominant syndrome that presents with diverse and complex clinical features and involves multiple human systems. TSC-related neurological abnormalities and organ dysfunction greatly affect the quality of life and can even result in death in patients with TSC. It is widely accepted that most TSC-related clinical manifestations are associated with hyperactivation of the mammalian target of rapamycin (mTOR) pathway caused by loss‑of‑function mutations in TSC1 or TSC2. Remarkable progress in basic and translational research has led to encouraging clinical advances. Although mTOR inhibitors (rapamycin/everolimus) demonstrate great potential in TSC management, two major concerns hamper their generalized application. One is the frequent manifestation of adverse events, such as stomatitis, infections, and menstrual disorders; and the other is the poor response in certain patients. Thus, indicators are required to effectively predict the efficacy of mTOR inhibitors. Herein, we have summarized the current utilization of mTOR inhibitors in the treatment of TSC and focused on their efficacy and safety, in an attempt to provide a reference to guide the treatment of TSC.

## Highlights

Hyperactivation of mammalian target of rapamycin (mTOR) is essential in the pathogenesis of tuberous sclerosis complex (TSC) and can serve as a therapeutic target.

mTOR inhibitors have shown considerable success in multiple clinical trials for the treatment of TSC, including neurological, pulmonary, cardiac, renal, and cutaneous phenotypes.

mTOR inhibitors are associated with adverse events, which should be considered during the management of TSC.

Indicators to predict mTOR inhibitor efficacy are required to select patients who are likely to benefit from such therapy.

## Introduction

As an autosomal dominant disorder, tuberous sclerosis complex (TSC) has an incidence of 1 in 6000–10,000 individuals [1]. It involves multiple human systems and presents with diverse and complex clinical features that result in different pathologies. For instance, TSC can lead to widely distributed hamartomas in multiple organs including the brain, lungs, heart, kidneys, and skin. Moreover, TSC can cause disabling neurological disorders such as epilepsy and TSC-associated neuropsychiatric disorders (TANDs).

**Fig. 1:**
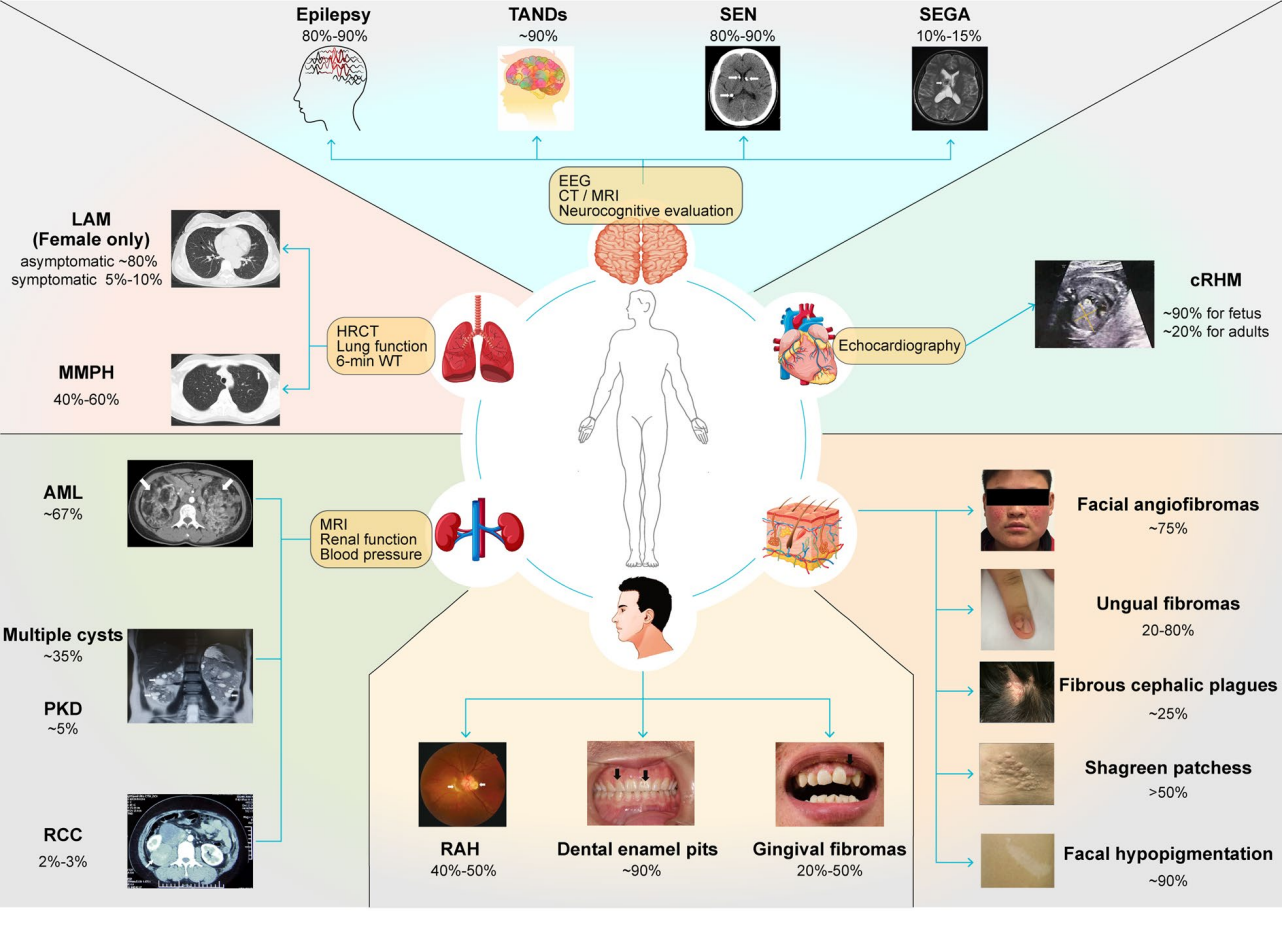
Clinical features of TSC are diverse and involve multiple systems. The most frequently affected systems, their associated lesions, and examination methods are shown. Percentages represent the approximate incidence in patients with tuberous sclerosis complex (TSC). *AML* angiomyolipomas; *cRHM* cardiac rhabdomyoma; *LAM* lymphangioleiomyomatosis; *MMPH* multifocal micronodular pneumocyte hyperplasia; *PKD* polycystic kidney disease; *RAH* retinal astrocytic hamartoma; *RCC* renal cell carcinoma; *SEGA* subependymal giant cell astrocytoma; *SEN* subependymal nodule; *TAND* tuberous sclerosis complex-associated neuropsychiatric disorder

These various manifestations with different penetrance are summarized in Fig. 1. Epilepsy is the most common disorder of the nervous system that affects 80%–90% of patients with TSC. It often starts in infancy and manifests as infantile spasms [2]. Although both subependymal nodules (SEN) and subependymal giant cell astrocytomas (SEGA) are space-occupying lesions growing beneath the ependyma, the probabilities of their occurrence are considerably different. Briefly, about 80%–90% of patients with TSC present with SEN, as determined using brain magnetic resonance imaging (MRI), whereas only 10%–15% of patients develop SEGA [3]. A recent growing concern among clinicians is the high incidence (approximately 90%) of TANDs in the TSC population, which include autism spectrum disorder (ASD), attention deficit hyperactivity disorder (ADHD), depression and anxiety, intellectual disability, and specific learning disabilities [4]. The most common morphology of TSC in the lung is lymphangioleiomyomatosis (LAM), a progressive condition characterized by cystic lung destruction with diffuse pulmonary infiltration of smooth muscle-like cells. On a cautionary note, it occurs in up to 80% of female patients without any clinical symptoms. Overt clinical symptoms including shortness of breath, fatigue and chest pain, or even respiratory failure, occur in only 5%–10% of the general population (including males) [5]. In contrast, multifocal micronodular pneumocyte hyperplasia (MMPH) affects males and females equally and has an incidence of 40%–60% [6]. MMPH causes small nodular lung deposits of type II pneumocytes that rarely progress to symptomatic disease. Cardiac rhabdomyoma (cRHM) is a common benign cardiac tumor in patients with TSC that is found in 90% of intrauterine fetuses; Unlike other TSC symptoms, most cRHMs subside spontaneously in early childhood [3]. However, it may also re-grow or occur de novo in adolescence, especially in girls [7]. Generally, approximately 20% adults still carried cRHM. Angiomyolipomas (AML) and simple multiple cysts, the two most common renal lesions of TSC, are mostly found in adult patients and accounts for 67% and 35% of cases, respectively [8–10]. More severe renal manifestations include polycystic kidney disease (PKD) and renal cell carcinoma (RCC), although they are also rarer with an incidence of 5% and 2%–3%, respectively [11, 12]. Skin lesions including facial angiofibromas (75%), ungual fibromas (20%–80%), fibrous cephalic plaques (25%), shagreen patches (> 50%), and focal hypopigmentation (90%) [3] develop in almost all patients with TSC [13]. Besides, retinal astrocytic hamartomas (RAH) were observed in 40%–50% of patients with TSC, especially in those carrying gene mutations [14]. Oral manifestations include gingival fibromas and dental enamel pits. The incidences are 20–50% and 90%, respectively [14]. The prognosis in individuals with TSC depends on the severity of their symptoms.

TSC is associated with significant involvement of human systems and can also lead to patient death due to accompanying symptoms. Specifically, the common causes of death include status epilepticus, bronchopneumonia, brain tumors, renal complications, pulmonary LAM, and neonatal heart failure caused by cRHM. Therefore, there is an urgent requirement to identify find safe and effective treatment approaches.

In the past, the management of TSC was unintegrated. For example, antiepileptic drugs were used only to treat epilepsy, surgery was used for SEGA and renal AML, and dermatological treatment was used in the management of skin lesions. With the gradual recognition that hyperactivation of the mammalian target of rapamycin (mTOR) signaling pathway plays an important role in the multisystem involvement of TSC, the efficacy of mTOR inhibitors for the treatment of TSC has been increasingly investigated (Table 1). mTOR inhibitors have shown great potential in the treatment of TSC. In addition to their routine use in the management of renal AML and SEGA, they also have a mitigating effect on other TSC-related manifestations such as epilepsy and skin lesions. However, few articles have systematically summarized the efficacy and safety of mTOR inhibitors for the treatment of TSC. A recent article systematically summarized only the adverse events (AEs) of everolimus based on the TOSCA cohort [15]. Herein, we discussed the role of the activation of the mTOR signaling pathway in the pathogenesis of TSC, and focused on both efficacy and safety of mTOR inhibitors in the treatment of various clinical manifestations of TSC. This has not been systematically reported and will provide an important and comprehensive reference for the clinical use of mTOR inhibitors in the context of TSC.

**Table 1:** Completed and ongoing clinical trials of rapamycin/everolimus on TSC in the past 15 years

Drug	Clinical trial accession no	Period	Population	Phase	No. of participants (age)	Status	Primary end point
**Brain**							
Rapamycin	NCT04987463	2021–2026	Infants with TSC	III	60 (≤ 16 weeks)	Recruiting	Efficacy, tolerability, and safety in seizures and TSC-related tumor volume
	NCT04595513	2020–2022	Infants with TSC and epilepsy	I/II	65 (≤ 6 months)	Recruiting	Epilepsy prevention
	NCT01929642	2013–2016	Patients with TSC and self-injury/autism	II	3 (2–30 years)	Completed	Efficacy and safety in autism
Everolimus	NCT01730209	2012–2016	Patients with TSC and TSC Related Cognitive Disability/TSC Related Autism/TSC Related Learning Problems	II/III	60 (4–15 years)	Unknown status	Efficacy and safety in autism and neuropsychological deficits
	NCT00411619	2007–2014	Patients with TSC and SEGA	I/II	28 (≥ 3 years)	Completed	Efficacy and safety in SEGA volume
	NCT01713946 (EXIST-3)	2013–2017	Patients with TSC-associated Refractory Seizures	III	366 (2–65 years)	Completed	Efficacy and safety as adjunctive therapy in refractory partial-onset seizures
	NCT01070316	2010–2016	Patients with TSC and epilepsy	I/II	20 (≥ 2 years)	Completed	Efficacy and safety in epilespy
	NCT00789828 (EXIST-1)	2009–2014	Patients with TSC and SEGA	III	117 (all ages)	Completed	Efficacy and safety in SEGA volume
	NCT01954693	2012–2018	Patients with TSC	II	48 (16–60 years)	Unknown status	Efficacy and safety in neurocognitive problems
	NCT02962414	2017–2027	Patients with TSC	III	206 (2–65 years)	Active, not recruiting	Long-term safety
	NCT02451696	2014–2017	Patients with TSC and epilepsy/focal cortical dysplasia	II	15 (2–40 years)	Completed	Effects of everolimus on brain mTOR activity and cortical hyperexcitability
	NCT01289912	2011–2014	Patients with TSC	II	52 (6–21 years)	Completed	Efficacy and safety in neurocognition
**Lung**							
Rapamycin	NCT02432560	2015–2021	Patients with LAM	NA	600 (≥ 18 years)	Active, not recruiting	Long term safety and efficacy in LAM
	NCT03150914	2018–2023	Patients with LAM	III	60 (≥ 18 years)	Recruiting	Efficacy in FEV1, DLCO and TLC
	NCT00414648	2006–2011	Patients with LAM	III	120 (≥ 18 years)	Unknown status	Safety and efficacy in LAM
Everolimus	NCT01059318	2010–2012	Patients with LAM	II	24 (≥ 18 years)	Completed	Safety and efficacy in LAM and change of VEGF-D
**Kidney**							
Rapamycin	NCT00490789	2005–2009	Patients with TSC and LAM	II	14 (18–65 years)	Unknown	Efficacy and safety in renal AML volume and FEV1
	NCT01217125	2008–2011	Patients with TSC and renal AML	IV	18 (≥ 10 years)	Completed	Efficacy and safety in renal AML volume
Everolimus	NCT00457964	2005–2013	Patients with TSC and LAM	I/II	36 (18–65 years)	Completed	Efficacy and safety in renal AML volume and FEV1
	NCT00790400 (EXIST-2)	2009–2015	Patients with TSC and LAM	III	118 (≥ 18 years)	Completed	Efficacy and safety in renal AML volume and FEV1
	NCT03525834	2018–2020	Patients with TSC and renal AML	IV	40 (≥ 18 years)	Completed	Efficacy and safety in renal AML volume
**Skin**							
Rapamycin	NCT03363763	2017–2022	Patients with TSC and facial angiofibromas	II	45 (2–21 years)	Recruiting	Safety and efficacy in cutaneous angiofibromas
	NCT03826628	2019–2021	Patients with TSC and facial angiofibromas	II/III	120 (6–65 years)	Recruiting	Safety and efficacy in facial angiofibroma
	NCT02634931	2015–2018	Tuberous Sclerosis Patients with TSC and skin lesions (angiofibroma, hypomelanotic macule or plaque)	III	94 (≥ 3 years)	Completed	Safety and efficacy in angiofibroma
	NCT01031901	2009–2011	Patients with TSC/NF1 and fibromatous lesions (angiofibromas or neurofibromas)	I	52 (≥ 13 years)	Completed	Safety in cutaneous fibromatous lesions
	NCT02635789	2015–2016	Tuberous Sclerosis Patients with TSC and skin lesions (angiofibroma, hypomelanotic macule or plaque)	III	62 (≥ 3 years)	Completed	Safety and efficacy in angiofibroma and other skin lesions
	NCT03140449	2013–2016	Patients with TSC and facial angiofibromas	III	52 (7–65 years)	Completed	Safety and efficacy in angiofibroma
	NCT01853423	2013–2016	Patients with TSC and facial angiofibromas	I	11 (3–45 years)	Completed	Efficacy, tolerability, and safety in facial angiofibroma
	NCT01526356	2012–2014	Patients with TSC and angiofibromas	II	179 (all ages)	Completed	Safety and efficacy in cutaneous angiofibromas

## Role of mTOR signaling pathway activation in the pathogenesis of TSC

**Fig. 2:**
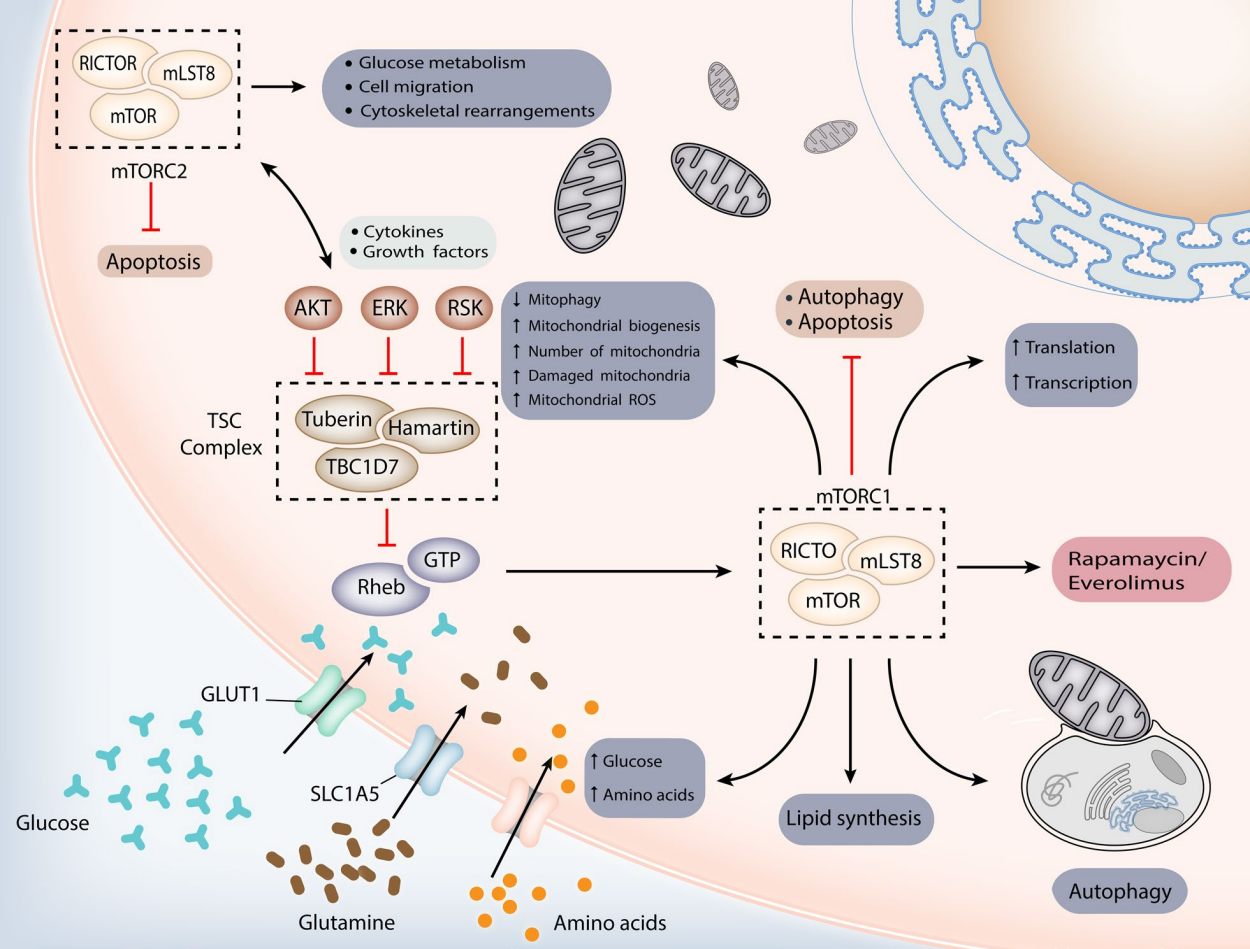
Dysfunction of the TSC complex causes mTORC1 hyperactivation through Rheb, which leads to metabolic and molecular changes. The TSC complex deactivates the RAS homolog enriched in brain (Rheb) by causing GTP to be cleaved from it. After stimulation by growth factor, the TSC complex is phosphorylated and its GTPase-activating protein activity is decreased. Similarly, dysfunction of the TSC complex is caused by loss-of-function mutations of *TSC1/TSC2* in the tuberous sclerosis complex (TSC). All of these factors activate Rheb to stimulate mammalian target of rapamycin (mTOR) complex 1 (mTORC1). mTORC1 directly regulates lipid, nucleotide, and protein synthesis to promote cell cycle progression and also inhibits autophagy. It ultimately causes excessive division and proliferation of cells to form hamartomas in multiple organs. *AKT* RACα serine/threonine-protein kinase; *ERK* extracellular-signal-regulated kinase; *GLUT1* solute carrier family 2, facilitated glucose transporter member 1 (also known as glucose transporter type 1, erythrocyte/brain); *mLST8* target of rapamycin complex subunit LST8; *mTORC2* mammalian target of rapamycin complex 2; *Raptor* regulatory-associated protein of mTOR; *RICTO* rapamycin-insensitive companion of mTOR; *ROS* reactive oxygen species; *SLC1A5* neutral amino acid transporter B(0); *TBC1D7* TBC1 domain family member 7

TSC is believed to develop from the pathogenic variants of the *TSC1* gene and more commonly the *TSC2* gene, which encode hamartin and tuberin, respectively [16, 17]. The encoded proteins physically interact with high affinity to form a heterotrimeric complex, termed the TSC protein complex, with the TBC1 domain family member 7 (TBC1D7) [18, 19]. After the discovery of *TSC1*, *TSC2*, and the TSC protein complex, substantial progress has been made toward understanding the pathogenesis of TSC. The most widely accepted mechanism is that *TSC1*/*TSC2* mutations inactivate the TSC protein complex, leading to a loss of inhibitory effect on the mTOR pathway, which mediates cell growth and metabolism in response to alterations in growth factors, cellular energy, and nutrient status (Fig. 2) [20]. Under normal conditions, growth factors stimulate the PI3K (phosphatidylinositol 3-kinase) and Ras–MAPK (mitogen-activated protein kinase) pathways, thereby inhibiting the TSC protein complex and activating mTOR signaling [21–23]. mTOR is a conserved serine-threonine protein kinase that regulates cell growth and proliferation through two distinct multimeric complexes: mTOR complex 1 and 2 (mTORC1 and mTORC2, respectively). mTORC1 integrates multiple upstream signaling pathways, including inputs from growth factors, amino acids, energy status, and cellular stressors such as hypoxia. It functions by phosphorylating two effector molecules, ribosome S6 protein kinase (p70S6K) and 4E-binding protein 1 (4E-BP1), to promote cell growth and proliferation. Specifically, the phosphorylation of p70S6K activates ribosomal protein S6, thereby increasing ribosomal recruitment and protein translation that is essential for cell expansion [24]. Meanwhile, the phosphorylation of 4E-BP1 can selectively promote the translation of mRNAs that support the survival of tumor cells during starvation [25–27].

Normally, mTORC1 can be activated by the RAS homolog enriched in brain (Rheb) by binding to guanosine-5′-triphosphate (GTP). A recent study has found that the TSC protein complex inhibits Rheb function via the GTPase-activating protein (GAP) domain near the carboxy terminal of tuberin [28]. Constitutively active Rheb hyperactivates mTORC1 in the absence of a functional TSC protein complex, leading to extensive metabolic reprogramming, including enhanced lipid, nucleotide, and protein synthesis and the inhibition of autophagy [18, 19, 29]. This reprogramming leads to vulnerabilities in induced cell death under specific conditions, such as in nutrient-deficient media. Moreover, the altered metabolism prone to anaerobic glycolysis is also one of the identified tumor hallmarks. Therefore, mTORC1 hyperactivation may partially explain the formation of TSC-associated solid tumors.

In the central nervous system, the mTOR pathway is involved in neuronal migration and cortical lamination, which are essential for the regulation of arborization of dendrites and determination of neuronal polarity in the early postnatal cerebral cortex [30]. mTORC1 hyperactivation and enhanced downstream activity induced by *TSC1*/*TSC2* mutations can lead to neuronal dysfunctions, as well as dysregulation or impairment of biological processes such as axon regulation and guidance, dendritic morphogenesis, synapse formation, and adaptation. Therefore, dysregulation of the mTOR pathway in patients with TSC also causes defects in these fundamental processes leading to abnormalities in neural circuit formation and activity-dependent plasticity, which in turn trigger epilepsy and TANDs. Specifically, upregulation of the mTOR pathway results in abnormal dendritic protein synthesis, reduced or deformed dendritic spines, and long-term depression mediated by alterations in the postsynaptic glutamate receptor [31–33]. Additionally, these synaptic abnormalities are considered to cause defects in learning, memory, and adaptability in patients with TSC having an ASD phenotype [34–36]. Moreover, changes in synaptic or cellular electrical properties may lead to abnormalities in neuronal excitability. The decrease in γ-aminobutyric acid (GABA)-ergic inhibition and increase in glutamatergic excitation are associated with higher epilepsy susceptibility in TSC populations and have the potential to elicit cognitive impairment [37–39].

With respect to TSC-associated skin lesions, the most frequently observed focal hypopigmentation has been suggested to be associated with mTOR. JóźWiak and Galus reported the inhibitory effect of mTOR hyperactivation on the microphthalmia-associated transcription factor in TSC, a master regulator of melanogenesis [40]. Hoet et al. proposed that mTORC1 inhibition promoted melanogenesis by increasing the transcription of melanogenic enzymes and the formation of mature melanosomes [41]. In a nutshell, hyperactivated mTORC1 suppresses melanogenesis, resulting in focal hypopigmentation in patients with TSC.

In summary, we have captured a trail of mTORC1 hyperactivation underlying the pathogenesis of TSC, confirming the critical role of this pathway in TSC, and highlighting its potential as a therapeutic target.

## mTOR inhibitors in the treatment of TSC

The currently recognized mTOR inhibitors against TSC include sirolimus and everolimus. Sirolimus, also known as rapamycin, was isolated in 1973 from soil samples in Easter Island [42]. Everolimus is a sirolimus derivative obtained by the addition of an ethyl ester group. Both compounds bind to and inhibit mTORC1, resulting in the allosteric dissociation of its cofactor rapTOR (TOR regulation-related protein, also known as FKB12), which is necessary for mTORC1 activity [43]. However, everolimus demonstrates better absorption, higher oral bioavailability, more rapid achievement of steady-state blood concentration after administration, and faster elimination after withdrawal compared with sirolimus [44–46]. Discoveries related to TSC pathogenesis have led to the hypothesis that the altered metabolism of cells due to mTORC1 hyperactivation may provide therapeutic opportunities. Specifically, the inhibition of mTORC1 using allosteric inhibitors restores metabolic homeostasis in abnormal cells, thereby reversing TSC-associated clinical manifestations [47]. Herein, we focus on the use of mTOR inhibitors in the treatment of TSC, especially the various related manifestations.

### Brain

#### Subependymal giant cell astrocytomas

In 2006, the first case series was published in which five patients with TSC having SEGAs were treated using open-label rapamycin. The tumor volume was significantly reduced in all patients. Given the regrowth of SEGA after drug discontinuation at the patient’s request, rapamycin treatment was restarted, which led to reshrinking of the tumor [48]. The mTOR inhibitor everolimus, a rapamycin derivative, shows better pharmacokinetics. The first prospective clinical trial of everolimus in the treatment of SEGA (n = 28) demonstrated a ≥ 30% volume reduction in SEGA in 21 patients and a ≥ 50% reduction in nine patients [49]. Based on these results, a complete clinical plan (EXIST-1) was developed to evaluate the efficacy and safety of everolimus in the treatment of TSC-related SEGA. In this multicenter, randomized, placebo-controlled phase III trial, 27 (35%) patients in the everolimus group experienced a ≥ 50% reduction in SEGA volume compared with no reduction in those in the placebo arm [50]. Furthermore, more patients achieved a ≥ 50% reduction in SEGA volume at long-term follow-up with blood concentrations of 5–15 ng/mL, and the efficacy of everolimus in reducing primary SEGA volume was maintained [51, 52]. Notably, SEGA rebound after everolimus discontinuation was observed in these clinical trials, suggesting that the treatment of TSC-associated SEGA requires continuous pharmacological intervention. Thus, in October 2010, the US Food and Drug Administration (FDA) approved everolimus to treat TSC-associated SEGA of all ages and recommended long-term treatment.

#### Refractory epilepsy

Refractory epilepsy is a common clinical manifestation in patients with TSC and can progress to status epilepticus, which is life-threatening [53]. TSC-associated epilepsy mostly responds poorly to traditional antiepileptics. Inspiringly, early clinical trials show that mTOR inhibitors reduce the frequency of refractory epilepsy and bring about tumor- and nodule-volume reduction [49, 51]. There was, however, a significant difference in the frequency of baseline epilepsy between the control and treatment groups, as most of these studies analyzed the control of epilepsy as a secondary endpoint. This aspect, combined with the fact that a large proportion of patients did not present with epilepsy at the beginning of treatment, greatly reduced the credibility of the results. Subsequently, researchers conducted a prospective clinical trial aimed at the treatment of refractory epilepsy with everolimus [54]. After 3 months of treatment, 17 (85%) participants reported a reduction in epilepsy frequency and 12 (60%) reported a ≥ 50% reduction in frequency from baseline. Additionally, a significant reduction in the duration of epilepsy episodes and improvements in parent-reported behavior and quality of life were reported. To further confirm this benefit, a phase III clinical trial (EXIST-3) was conducted in a large sample population to assess the efficacy and safety of everolimus as adjuvant therapy in patients with TSC having refractory focal-onset epilepsy [55]. Briefly, 366 participants aged 2–65 years were randomly divided into a placebo arm (n = 119), low-dose everolimus arm (blood concentration: 3–7 ng/mL; n = 117), or a high-dose everolimus arm (blood concentration: 9–15 ng/mL; n = 130). The primary endpoint was the proportion of patients achieving a ≥ 50% reduction in epilepsy frequency. The response rates were 15.1%, 28.2%, and 40.0% in the placebo, low-dose everolimus, and high-dose everolimus arms, respectively, which rigorously demonstrated the excellent remission efficacy of everolimus in TSC-related refractory epilepsy. Meanwhile, this was the first evidence-based study to propose the superiority of high-dose mTOR inhibitors over the low-dose ones. Nonetheless, it was found that patients treated with low-dose everolimus may achieve outcomes similar to those treated with high doses after prolonged treatment. Thus, this clinical trial facilitated the progression of the FDA approval of everolimus as an adjuvant treatment for TSC-associated epilepsy. Clinical practice has demonstrated that everolimus can reduce the dose of antiepileptic drug combinations in the treatment of TSC-associated epilepsy. Furthermore, everolimus can be used as monotherapy to control epilepsy in certain patients.

Although everolimus demonstrates strong efficacy, patients with poor economic conditions tend to choose the more affordable rapamycin as routine medication. Several researchers have investigated the efficacy of rapamycin in controlling TSC-associated epilepsy and found a 41% decrease in epilepsy frequency compared with the standard-of-care treatment in pediatric patients [56]. More recently, a Chinese cohort with 91 pediatric individuals with TSC reports a response rate of 78.0%, and even 47.2%, of patients achieved epilepsy-free [57]. These results suggest that rapamycin can be used as an alternative to everolimus in the adjuvant treatment of TSC-associated epilepsy when necessary.

#### White matter diffusion

Studies have described abnormalities in normal-appearing white matter (NAWM) in TSC based on diffusion tensor imaging (DTI), which included decreased fractional anisotropy (FA) or increased mean diffusivity measures within tubers surrounding the subcortical white matter [58–60]. Subgroup analyses based on the DTI data from phase I/II clinical trials evaluating the efficacy of everolimus in the treatment of SEGA reveal significant changes in FA and radial diffusion rate after everolimus treatment in patients with TSC [61]. These findings indicate a pharmacological modification over the TSC genetic defects in the brain and provide the possibility that mTOR inhibitors may have a therapeutic effect on TSC-associated mental ailments.

#### TSC-associated neuropsychiatric disorders

The treatment of TANDs has always been challenging due to their complex pathogenesis. In an open epilepsy trial, Krueger et al. reported a statistically significant improvement in adaptive social behavior, behavioral problems, and insecurity/anxiety compared with the corresponding presentation at baseline. The quality-of-life assessment for epileptic children revealed similar improvements in many areas, including attention, behavior, social interaction and activities, and the overall quality of life [54]. However, it is uncertain whether these changes are secondary to or independent of epilepsy control.

Therefore, a prospective phase II study was conducted to evaluate the efficacy of everolimus in treating TANDs. A total of 47 children age 6–21 years were randomized in a 2:1 ratio of everolimus:placebo for 6 months [62]. It was found that everolimus treatment failed to improve TANDs in this study. A recent study enrolled 60 children aged 4–17 years with TSC and IQ < 80, learning disabilities, special schooling or autism, and no refractory epilepsy, to investigate whether everolimus (blood concentration: 5–10 ng/mL) could improve intellectual disability, autism, and other neuropsychological deficits [63]. Similarly, no positive results were found. We cannot arbitrarily conclude that everolimus does not mitigate TANDs, as some clinical trials are still underway and have not yet published their findings. For example, the trial of everolimus and neurocognition in TSC (NCT01289912), the TRON study (NCT01954693), the RAPT study (NCT01929642), and the phase II/III Dutch RAPIT study (NCT01730209) are still ongoing.

### Lung

#### Lymphangioleiomyomatosis

As a secondary endpoint, a pilot study of rapamycin for AML showed improvement in spirometric measurements and persisted gas trapping in some patients with LAM [64], indicating the potential of mTOR inhibitors in the treatment of TSC-associated LAM. Thereafter, a randomized placebo-controlled trial of rapamycin was conducted in adult patients with moderate LAM, and significant remission of the loss of lung function and an improvement in forced vital capacity, quality of life, and functional performance was reported. The lung function deteriorated again and paralleled that in the placebo group after the discontinuation of rapamycin [65]. Therefore, lifelong continuous treatment with rapamycin is recommended. Another similar study reported an improvement and stabilization of lung function and a reduction in the extent of chylous effusions and LAM after treatment with rapamycin [66]. Based on these results, the FDA approved rapamycin in 2015 for the treatment of TSC-associated LAM.

Considering the more optimal pharmacokinetics of everolimus, a study explored the efficacy of everolimus in 24 adult women with LAM and found that lung function and exercise capacity could be improved by everolimus treatment [67]. In general, mTOR inhibitors retard the deterioration in lung function in most women with LAM; however, whether they can prevent the progression of LAM is still unclear.

#### Multifocal micronodular pneumocyte hyperplasia

As MMPH is rarely clinically symptomatic, there are only a few studies that have focused on the medical treatment of MMPH. A recent study first reported a significant shrinkage of MMPH was achieved in a 25-year-old man after treatment with mTOR inhibitor [68]. Although this is only a case report, the findings lay a foundation for follow-up studies focusing on the therapeutic effects of mTOR inhibitors in MMPH.

### Heart

#### Cardiac rhabdomyoma

Despite the generally favorable outcomes and spontaneous regression, multiple or large cRHMs may result in heart failure or cardiac arrhythmias that require intervention. A meta-analysis summarized the use of mTOR inhibitors in the treatment of cRHM in pediatric patients, most of which were neonates [7]. It reported that the clinical symptoms improved in 90.9% of patients after treatment and the size of cRHM was reduced by 95.1%. Although some cRHMs recurred after withdrawal of the mTOR inhibitor, clinical symptoms were mostly not observed. However, it is important to note that most of the included studies were case reports. A lack of randomized or large cohort studies reduces the validity of the estimated effects. Moreover, it is difficult to determine whether the reduction was due to the efficacy of the mTOR inhibitor or spontaneous regression, given the inherent nature of cRHM. Findings from a recent case series provide the answer. This case series evaluated the tumor regression in four neonates with cRHM after everolimus treatment and compared it with natural regression. Tumor regression after everolimus treatment was nearly 12 times faster compared with the control, confirming the efficacy of everolimus in cRHM [69]. Thus, everolimus may be a suitable treatment option for patients who are symptomatic and are at potential risk for serious cardiac events due to cRHM-related obstruction [70]. More definitive evidence is needed for asymptomatic patients to establish that everolimus use confers significant benefits.

### Kidneys

#### Renal angiomyolipomas

Renal AML contributes to chronic kidney disease (CKD) and intrarenal hemorrhage and is a common cause of TSC-related mortality. The traditional treatment of renal AML, including embolization or (partial) nephrectomy, has significant disadvantages. Collateral damage to normal renal tissues may aggravate the risk of renal dysfunction [71, 72] and lead to a high risk of postembolization recurrence [72–74]. Therefore, new therapies are warranted. With an increase in the understanding of the pathogenesis of TSC, several attempts have been initiated for the use of mTOR inhibitors in the treatment of renal AML. The clinical response of renal AML to an mTOR inhibitor was first reported in 2006. AML was significantly reduced in a 19-year-old patient after treatment with rapamycin and his tumor relapsed after drug discontinuation. Upon reinitiating rapamycin, the tumor shrank again [75]. The CAST trial was the first prospective clinical trial that evaluated the therapeutic effects of rapamycin in 25 patients with AML aged 18–65 years. The blood concentration was controlled in 1–15 ng/ml, which was adjusted according to the response. After 12 months of treatment, the mean AML volume decreased to 53.2% ± 26.6% of the baseline value. However, the mean volume increased to 85.9 ± 28.5% of the baseline value at the 12th month after rapamycin withdrawal [64], warranting continued treatment. Similar results have been reported by several multicenter phase II clinical trials [76–79]. Moreover, most shrinkage occurred during the first year of treatment before the stabilization of AML in continued therapy.

A randomized phase III trial (EXIST-2) with a large sample size was initiated after the discovery of everolimus to evaluate its efficacy in the treatment of TSC-associated AML for adult patients [80]. In this trial, the response rate of AML to everolimus was 42% compared with 0 with placebo, demonstrating the remarkable efficacy of everolimus in treating AML. Another study focusing on the short-term use of everolimus reported that the mean reduction in AML volume was 56.47 ± 23.32% within 12 weeks, and that drug discontinuation resulted in regrowth [81]. Moreover, the efficacy and safety of everolimus in the treatment of renal AML have also been confirmed in patients aged < 18 years with the blood concentration of 5–15 ng/mL [82].

Considering the encouraging efficacy of mTOR inhibitors, the International Tuberous Sclerosis Complex Consensus Conference held in 2012 recommended mTOR inhibitors as the first-line treatment for renal AML with a diameter ≥ 3 cm, even if patients did not present with any clinical symptoms [71].

#### Cystic disease

As the *TSC2* and *PKD1* genes are adjacently located, *TSC2* mutation is sometimes accompanied partially or completely by *PKD1* mutation, which can cause PKD, also known as the contiguous gene syndrome. The renal phenotype in these patients tends to be more severe with premature progression to renal failure [11, 83]. Besides, some TSC patients suffer simple cytic disease without PKD gene mutation. However, safe and effective therapies are currently unavailable for TSC-associated renal cystic disease, although a study has reported that rapamycin may have some therapeutic effect. This study enrolled 15 pediatric patients with known renal cystic disease and TSC. However, these patients have not been detected for PKD gene, so it is impossible to distinguish between the contiguous gene syndrome and simple cytic disease in TSC. Generally, for TSC-associated renal cystic disease, the results showed a decrease in the sum of cyst diameters of 15 patients, a decrease in the total cyst volume in 14 patients, and a 72.1% decrease in the cyst number [84]. The sample size of this study is relatively small. Therefore, whether mTOR inhibitors are effective in TSC-associated renal cystic disease requires further investigation.

#### Renal cell carcinoma

Treatment strategies for TSC-associated RCC are similar to those used for general RCC and include surgery, chemoradiotherapy, and immunotherapy. Everolimus is currently approved for the treatment of advanced RCC after failure of initial therapy with tyrosine kinase inhibitors. However, to date, there is only one case report that shows that a 47-year-old patient with TSC-associated metastatic RCC continued to benefit from everolimus over a 2-year follow-up [85]. Thus, additional evidence from cohort studies is required to obtain further therapeutic insights into TSC-associated RCC.

### Skin

#### Facial angiofibromas

TSC-associated facial angiofibromas have previously been treated using laser surgery, cryotherapy, resurfacing, or similar approaches. However, pain, scarring, and lesion recurrence cannot be avoided [86–88]. Initial findings from EXIST-1 and EXIST-2 reported a partial response of skin lesions during treatment with systemic mTOR inhibitors [50, 80], which was further confirmed based on the long-term follow-up of oral everolimus [89]. However, treatment with systemic mTOR inhibitors is associated with serious AEs; thus, they are not approved solely for the treatment of skin lesions. In 2012, the first study of topical rapamycin for the treatment of TSC-associated facial angiofibromas demonstrated considerable efficacy and safety in 28 patients aged > 13 years (blood concentrations < 1.0 ng/mL), wherein 73% of patients treated with topical rapamycin versus 38% treated with placebo reported a subjective improvement in angiofibromas [90]. Similar results were obtained in another left–right comparative study, which enrolled 11 patients aged < 10 years [91]. Thus, the 2012 International Tuberous Sclerosis Complex Consensus Conference included topical rapamycin into their treatment recommendations for angiofibromas [71]. Subsequently, several clinical trials and meta-analyses reported similar results and proposed an optimal drug concentration of 0.2% [92–95]. Besides, more studies also reported that topical rapamycin is safe and effective in pediatric patients. Younger children tend to respond better, which may be attributed to the more prominent vascular components of newly developed facial angiofibromas, as vascular components elicited a better response to rapamycin than older facial angiofibromas [96, 97]. Therefore, early treatment initiation is recommended. A randomized clinical trial recently demonstrated rapamycin-calcitriol ointment as a clinically beneficial and safe treatment option for the treatment of facial angiofibroma [98]. The advantages include a more rapid improvement of erythema, a more effective reduction in papule elevation, and longer control of the condition after drug discontinuation compared with that achieved using rapamycin alone.

#### Focal hypopigmentation

TSC-associated focal hypopigmentation is resistant to conventional treatment in dermatology. Previous studies have reported that rapamycin upregulates microphthalmia-associated transcription factor in B16 and MNT-1 melanoma cells, implying that rapamycin may be potentially effective in treating focal hypopigmentation [99, 100]. Subsequent to a case report showing focal hypopigmentation recovery in two pediatric patients with TSC after treatment with topical rapamycin [101], a prospective trial of topical rapamycin (drug concentration: 0.2%) in the treatment of focal hypopigmentation was conducted, which enrolled 6 patients aged 3–33 years. The findings not only demonstrated the efficacy and safety of topical rapamycin for TSC-associated focal hypopigmentation but also confirmed that its efficacy was due to improved melanogenesis in TSC melanocytes [102]. However, this study had a small sample size of six patients and more evidence is required to further validate its efficacy and safety and provide better clinical guidance.

## Adverse effects

Stomatitis is the most common AE of mTOR inhibitors with an incidence of approximately 70% [50, 55, 80, 103]. However, most stomatitis events were grade 1/2 and self-limiting, which did not affect the continuation of the mTOR inhibitor. Maintenance of good oral hygiene, using a buffer mixed with mTOR inhibitor, and gargling with sucralfate or topical steroids can suitably control mild stomatitis [43]. Stomatitis may be severe in some cases, but is generally reversible by dose reduction or temporary discontinuation [104].

Infection is another common AE of mTOR inhibitors that is characterized by pyrexia, diarrhea, nasopharyngitis, and upper respiratory tract infections [46, 51, 77]. Although these AEs are often mild, severe cases have also been reported. For example, four patients died during the EXIST-3 study, among whom two succumbed to infection (pneumonia and septic shock) [55]. In addition to the broad immunosuppressive phenotype resulting from the use of mTOR inhibitors, another important reason is that patients often have difficulty expectorating respiratory secretions due to concomitant intellectual disability or pseudobulbar palsy, thereby increasing the risk of respiratory infections [105, 106]. Thus, mTOR inhibitors can be continued in a clinical setting for mild infections; however, they should be discontinued if the severity of the infection increases.

Of the non-infectious AEs, menstrual irregularity is the most common. Among women ≥ 13 years of age who received everolimus, 3 (37.5%) in EXIST-1 and 7 (13.5%) in EXIST-2 developed secondary amenorrhea [50, 80]. Half of them recovered spontaneously whereas the other half resumed normal menstruation after progesterone administration. A meta-analysis reported that 43 (38.4%) patients experienced at least one menstrual irregularity. Amenorrhea and menstrual irregularity occurred in 24.1% and 17.0% of patients, respectively, and seven patients experienced grade 3/4 amenorrhea [107]. Thus, it is necessary to monitor this AE in female patients of child-bearing age.

There are some less common AEs apart from the ones described. First, skin toxicity has been reported during therapy with mTOR inhibitors and typically presents as maculopapular or acneiform rashes [104]; its incidence in EXIST-1 and EXIST-2 was 12% and 22%, respectively [50, 80]. These skin reactions are also usually mild and self-limiting. Topical steroids are prescribed when necessary. Second, rapamycin and everolimus can significantly increase serum lipid levels by reducing lipoprotein lipase activity [104, 108]. Mild/moderate hyperlipidemia can be treated solely by dietary intervention, whereas severe hyperlipidemia may require a dose reduction of rapamycin and everolimus and/or additional hypolipidemic medications. Lastly, hyperglycemia and myelosuppression may occur during mTOR inhibitor therapy [104]. Hyperglycemia is not clinically significant in most patients; however, strict glycemic control is required in patients with pre-existing diabetes mellitus. Myelosuppression caused by mTOR inhibitors is usually minor and reversible. If it occurs, the dosing of mTOR inhibitors should be adjusted, or these drugs should either be completely withdrawn or replaced. Proteinuria is another important mTOR inhibitor–associated AE [109], which has previously been reported in small single-arm studies with sirolimus [77, 78], A recent large sample study reported that everolimus can increase the incidence of proteinuria,which was mostly Grade 1/2 in severity, with Grade 3 proteinuria reported in only two patients [110]. In practice, if proteinuria is > 1 g/day and rising progressively, the dose of mTOR inhibitors should be adjusted (reduced or temporarily suspended until proteinuria is < 1 g/day).

In general, the management of AEs mainly includes dose reduction or temporary cessation [111]. Mild (grade 1/2) AEs usually do not require dose adjustment. When a reduction is required, a dose of 50% of the original dose is recommended. For dose reduction below the minimum available intensity, alternate-day administration should be considered. If an AE persists or relapses, the treatment should be interrupted for 3–14 days (or until the AEs drop to ≤ grade 2) and restarted at a lower dose. Dose interruption/reduction can help ameliorate AEs while allowing for continued therapeutic benefits. For example, dose interruption/reduction was necessary for 71% of patients in EXIST-2, wherein approximately one-third of the dose was maintained below the initial dose of 10 mg/day [80].

## Indicators to predict the efficacy of mTOR inhibitors

Despite the promising efficacy of mTOR inhibitors in multiple clinical phenotypes associated with TSC, there is still a subset of patients who do not respond well to mTOR inhibitors. Therefore, studies have been conducted to explore the indicators based on predictive value to screen and identify patients who may benefit from mTOR inhibitor therapy.

### Computed tomography (CT) value of AML

In our previous study, we found that renal AML volume and baseline mean CT value were important factors affecting the short-term volume response of AML to mTOR inhibitors. Further, statistical analysis revealed that a high CT value (≥ − 7 Hu) may imply a better response [112]. AML with low CT value and large volume often has a very high fat count. Because fat is trapped in poorly perfused cells that become more ischemic following mTOR inhibitor therapy, the response is poor. However, any potentially dangerous epithelioid/vascular components contained within them do shrink. Therefore, we recommend mTOR inhibitor therapy for patients with renal AML who exhibited a high CT value.

### Vascular endothelial growth factor (VEGF)-D

Studies have shown a significant correlation between the vascular growth-promoting cytokine VEGF-D and renal AML size. Both exhibited a similar decreasing trend during continuous rapamycin treatment [77, 79, 80]. Taken together with our previous finding that renal AML volume affects the response to mTOR inhibitors, it is reasonable to speculate that high serum VEGF-D levels may imply a better response to mTOR inhibitors. Additionally, it was found that serum VEGF-D levels were related to the severity of LAM. Patients with higher serum VEGF-D levels had more significant improvements in FEV1 and FVC after rapamycin treatment, and serum VEGF-D levels decreased with an improvement in lung function [113]. These results suggest that serum VEGF-D is a useful marker not only in predicting efficacy but also in monitoring the response during treatment with mTOR inhibitors.

### Genotype

An increasing number of patients with TSC have undergone *TSC1/TSC2* genetic testing since the presentation of the genetic diagnosis of TSC at the 2012 International Tuberous Sclerosis Complex Consensus Conference. In addition to the diagnostic value, increasing attention has been paid to genetic testing in guiding therapy. However, no apparent associations between genotype and treatment response were found in the EXIST-1 and EXIST-2 trials [114].

Currently, the management of TSC is systematic. The discovery of mTOR pathway upregulation in TSC represents a potential opportunity for the targeted therapeutic strategy to control TSC-associated manifestations. Existing clinical trials have demonstrated that many patients with TSC can benefit from mTOR inhibitor therapy. Despite this breakthrough, several problems remain to be resolved. First, tumors regrow after treatment discontinuation, which necessitates lifelong therapy. Hence, there is an urgent requirement for new therapies that can permanently kill tumor cells and eliminate the need for continuous therapy. Second, although some molecular markers have been found to predict treatment efficacy, other indicators should be explored from the point of precision therapy. Lastly, although the AEs of mTOR inhibitors are mostly mild, some severe AEs have been reported that deserve attention [115]. Alternatives are required for patients who do not tolerate mTOR inhibitors.

## Conclusion

mTOR inhibitors are effective in controlling TSC-associated clinical manifestations, implying a bright future for patients with TSC. Although several issues exist, we can be optimistic that better therapeutic strategies are underway.

## Data Availability

Not applicable.
